# Abnormally Large Superior Strait, a Complication of Labor

**Published:** 1876-05

**Authors:** J. Suydam Knox

**Affiliations:** No. 16 Loomis St.; Chicago


### EXAMINER.

Vol. XXXIII. — MAY, 1876. — No. 5.

### Original Communications.

### ABNORMALLY LARGE SUPERIOR STRAIT, A COMPLICATION OF LABOR.

By J. SUYDAM KNOX, M.D., Chicago.

At first sight such a statement would appear a contradiction, for with apparently greater facility of exit, one would expect more rapid delivery. The following six cases, however, while too few to make the statement a truism, go far to establish its correctness.

The six patients referred to below were all ladies simply exercising household supervision, and doing no manual labor. They were also all primiparæ.

Case 1. Mrs. T——, æt. 24, a tall, slender blonde. Labor commenced Aug. 11, 1871. Vaginal examination revealed the head presenting, enclosed in the womb, and resting upon the perineum; os tincæ moderately far back towards coccyx, and undilated; uterine contractions energetic, frequent and regular, and exceedingly painful. No progress being made during several hours, nauseating doses of tartarized antimony were administered, and subsequently ⅛ gr. morphia hypodermically. Under the influence of the opiate, the uterine os was brought forward and dilated, and the membranes ruptured. The first stage of labor had lasted twenty-four hours, the second stage was completed in two hours.

In this case there was but little obliquity of the womb. The os tincæ was dilatable but did not dilate. No bag of waters formed.

Case 2. Mrs. H——, æt. 23, undersized, slender blonde. Labor commenced about term, July 19, 1872, caused by the unexpected breaking of the waters during sudden fright. Vaginal examination revealed the uterus very low down in pelvis, os tincæ far back in hollow of sacrum; vertex presenting; patient hysterical, with feeble and irregular uterine contractions. Administered ⅛ gr. morphia hypodermically. After a sleep of six hours uterine expulsive pains came on regularly and energetically, and continued so for seven hours, when delivery was accomplished. Child weighed 4½ pounds.

In this case, owing to accident, there was no first stage of labor. The second stage was evidently prolonged through seven hours, by the low position of the womb. The uterine os, though flaccid, would not dilate, and the strong pains seemed to have no expulsive power. The use of forceps was forbidden.

Case 3. Mrs. K——, æt. 28, slender, medium-sized blonde. Examination of urine thirteen days before labor showed no albuminuria. Labor commenced Oct. 8, 1873. Vaginal examination revealed uterus enclosing fœtal head resting upon perineum, os tincæ easily reached and dilating. Labor progressed favorably for four hours, when, without warning, a severe convulsion occurred; patient immediately bled about twenty ounces, and delivered with forceps. One-half grain morphia then injected hypodermically. Made a good recovery.

In this case, the patient was extremely small during pregnancy, even at term giving scarcely any external evidence of her condition. This was due to the low position of the womb and the absence of any obliquity of the organ. Had convulsions not occurred, delivery would have soon been accomplished naturally. The extreme and unnatural descent of the womb, however, undoubtedly caused pressure upon the ureters, and produced the uræmia and convulsion. Albuminuria existed for several days after labor, and the urine was loaded with uric acid crystals.

CASE 4. Mrs. McD——, æt. 25, a full-sized brunette. Labor commenced April 7, 1875. Vaginal examination revealed fœtal head within uterus resting upon perineum, os tincæ not to be felt; uterine contractions strong, frequent, regular, and very painful throughout labor. Persistent effort at last engaged two fingers in the uterine os, which was brought forward between the pains. As soon as sufficiently dilated, the membranes were ruptured and the labor completed. First stage of labor, eight hours; second stage, two hours.

In this case, the same conditions, of dilatable but undilating os, and non-expulsive though severe pains, existed.

CASE 5. Mrs. S——, æt. 21, tall, slender brunette. Was called in ten days before labor on account of extreme general dropsy. Examination of urine revealed small amount of albumen. Prescribed 3 drachms daily, of bitartrate of potassa largely diluted, and full doses of quinine and iron. Labor commenced May 3rd, 1875; at that time dropsy much diminished and no albuminuria. Vaginal examination revealed the same conditions as in Case 4, and the labor was in every way similar, the first stage lasting twelve hours, and second stage two hours.

In this patient, we have the conditions existing in Cases 3 and 4, viz.: dilatable but undilating os; strong but non-expulsive pains; and compression of the ureters, producing dropsy and albuminuria.

CASE 6. Mrs. E——, æt. 26, medium-sized, slender brunette. Was called to see this patient thirteen days before labor on account of extreme general dropsy; her face, neck, hands and lower extremities were largely œdematous, and the genitalia so swollen as scarcely to admit of vaginal examination. For the few last days had suffered from dizziness and momentary attacks of blindness; was also asthmatic; the urine a few days before had been tested and found normal; some, now drawn off, was found to be largely albuminous. Prescribed iron and quinine, and drinks of solut. bitartrate of potassa. Nov. 30, 1875, labor commenced; at this time the dropsy, especially of the genitalia, was still enormous and the urine albuminous. Vaginal examination discovered the fœtal head, within the womb, resting upon the perineum, and the os tincæ far back in the hollow of the sacrum. As labor pains had already existed twenty-four hours and patient was restless and nervous, I administered, per orem, 45 grs. chloral hydrate; assisted by its influence the uterine os was brought forward. The fœtal head was then allowed for six more hours to drive upon the perineum, to expel, if possible, the œdema; a slight convulsion then occurred; 30 grs. additional hydrate of chloral were administered, the membranes ruptured and forceps applied; a living child was delivered, and the perineum lacerated to the sphincter ani. For six days subsequently there was retention of urine, requiring the daily use of the catheter. Duration of first stage of labor, thirty hours.

In this case, the vilest I ever attended, there were the same conditions of dilatable but undilating os, and severe and painful but non-expulsive uterine contraction.

In reviewing these cases, two things are noticeable:

1. The increased length of the first stage of labor.

2. The albuminuria which complicated one-half of them.

1. The prolongation of the first stage of labor is traceable directly to the non-expulsive character of the pains, and the non-dilatation of the os tincæ; and indirectly to the abnormally large diameters of the superior strait.

Without entering into particulars, it may be said of a labor pain, during a strictly normal labor, that the circular uterine fibres by their contraction diminish the transverse diameters of the womb, while the longitudinal fibres by synchronous contraction diminish its long diameter. In this way the waters are forced down into the cervix, upon the protruding membranes, to dilate the external os, at the same time that the womb, fixed upon the body of the child, tends to draw itself up over the fœtal head, thus making the pain expulsive. In other words, the contractions of the circular fibres are mostly compressive and tend to drive out the waters, while the contractions of the longitudinal fibres, using the fundus as a fulcrum, are mostly expulsive and tend to drive out the child. Both tend, during the first stage of labor, to dilate the os tincæ, and a successive repetition of them finally ruptures the membranes and completes this stage of labor.

In the class of cases under consideration, however, a different condition of things exists. The womb has descended low into the true pelvis. When now a uterine contraction occurs, the longitudinal fibres caught midway between the fœtal head and the pelvic brim are strongly compressed and fail to contract. Thus the long diameter of the womb is not diminished, and the pain is not expulsive. This annular compression of the uterine walls is extremely painful, and thus arises another factor in restraining the expulsive force of the contractions. In the six cases reported, each patient complained of agonizing pain with each uterine contraction, located principally in the groins and back. It was impossible to bear down, or in any way to assist by voluntary effort the labor of the womb.

The non-dilatation of the os tincæ can now readily be accounted for. The longitudinal uterine fibres which should expand it over the fœtal head are powerless, at the same time the circular fibres are active and render it rigid with each throe of parturition. Still further, two mechanical obstacles effectively prevent the formation of a bag of waters. The fœtal head, accurately fitting the superior strait, lined, as it is, with the muscular tissues of the womb, prevents all egress of amniotic waters, and holding the os tincæ firmly against the perineum or in the hollow of the sacrum permits no protrusion of the membranes. These are the reasons why, in all the cases above recorded, the os was invariable in its rigidity and small orifice during the uterine contractions, however dilated and relaxed it may have been in the intervals between the spasms.

With such results, it is not difficult to go back to the remote cause, and see how an abnormally large pelvic brim can render the first stage of labor tedious and painful.

2. I take it, however, that there is a much graver complication of labor growing out of this condition, namely: albuminuria, with or without general œdema.

Of the six cases reported, 50 per cent. had albuminuria and 33⅓ per cent. eclampsia. The per centage is suspiciously large. It is, however, more noticeable, when it is considered that these six cases are the only ones of the kind in 185 recorded cases of labor, and that in these 185 cases there were but six cases of puerperal eclampsia (a large proportion). In other words, one-third, or 33⅓ per cent., of all the cases of eclampsia in 185 labors, could apparently be traced to an abnormally large superior strait, and a low position of the gravid womb in the true pelvis. I know that the number of labors is too small to furnish accurate and positive conclusions, but as before said, the percentage is suspiciously large. One could reasonably suppose that the position of the womb had much to do with the albuminuria and convulsions.

If the commonly received opinion be true, that renal congestion be due to direct compression of the kidneys or of the renal veins, one would suppose that, with a womb so low in the pelvis, such pressure would be at a minimum, and would reasonably expect a decreased tendency to albuminuria—a conclusion exactly the reverse of true. Again, in all pregnant women there is always a certain average amount of pressure from the gravid womb upon the abdominal contents. Why then should one escape albuminuria more than another? The question becomes more pertinent when we find that those with uteri abnormally large at term, have no special tendency to nephritic congestion, unless from downward pressure œdema is superadded. Indeed, to my mind it is doubtful if nephritis often originates in this manner. The spinal column is so prominent in the abdominal cavity, that it must receive to a large extent the full force of the backward shove of the uterus; while the kidneys, spared through the globular shape of the womb, and their own encasement in adipose tissue, lie safely in the hollows each side of the spine. Further, the inferior vena cava lies to the right of, and below, the prominence of the spine. Thus the right renal vein is safe from pressure, while the left, crossing the spine above the summit of its forward lumbar curve, can scarce be compressed by the convex fundus, owing to the obliquity of the womb forwards.

I prefer to think, that albuminuria is oftenest caused by pressure upon the ureters; either as they escape over the brim of the pelvis, or as they discharge themselves into the bladder, thus obstructing the outflow of urine, and starting backward the train of symptoms whose frequent culmination is eclampsia. The comparative rarity of puerperal convulsions, and their special predilection for primipara, can thus, I think, best be accounted for.

The conditions most favorable for such compression are found in the six cases reported, and while these cases are but about three per cent. of the recorded labors from which they are taken, they furnish over thirty-three per cent. of the cases of eclampsia.

Finally, the six reported cases were all primiparæ. In such, similar conditions exist for the compression of the ureters beyond what is found in multiparæ, but from a different cause. It would be a mistake to suppose that the resistance to the growing womb of the firm and unrelaxed abdominal walls was only, in direction, from before backward. It is rather from every side inward and downward, thus keeping the womb wedged into the brim of the pelvis, and directly pressing upon the ureters as they escape into that cavity.

No. 16 Loomis St.

